# Core–Shell Structured Carbon Nanofiber-Based Electrodes for High-Performance Supercapacitors

**DOI:** 10.3390/molecules28124571

**Published:** 2023-06-06

**Authors:** Peizhi Fan, Jie Wang, Wenfei Ding, Lan Xu

**Affiliations:** 1 National Engineering Laboratory for Modern Silk, College of Textile and Engineering, Soochow University, Suzhou 215123, China; 20205215011@stu.suda.edu.cn (P.F.); 20215215113@stu.suda.edu.cn (J.W.); 20225215012@stu.suda.edu.cn (W.D.); 2 Jiangsu Engineering Research Center of Textile Dyeing and Printing for Energy Conservation, Discharge Reduction and Cleaner Production (ERC), Soochow University, Suzhou 215123, China

**Keywords:** electrospinning, NiS, hydrothermal process, specific capacitance, asymmetric supercapacitor

## Abstract

The combination of multiple electrode materials and their reasonable structural design are conducive to the preparation of composite electrodes with excellent performance. In this study, based on carbon nanofibers grown with Ni(OH)_2_ and NiO (CHO) prepared by electrospinning, hydrothermal growth, and low-temperature carbonization, five transition metal sulfides (MnS, CoS, FeS, CuS, and NiS) were hydrothermally grown on their surfaces, exhibiting that CHO/NiS had the optimal electrochemical properties. Subsequently, the effect of hydrothermal growth time on CHO/NiS revealed that the electrochemical performance of CHO/NiS-3h was optimal, with a specific capacitance of up to 1717 F g^−1^ (1 A g^−1^), due to its multistage core–shell structure. Moreover, the diffusion-controlled process of CHO/NiS-3h dominated its charge energy storage mechanism. Finally, the asymmetric supercapacitor assembled with CHO/NiS-3h as the positive electrode demonstrated an energy density of 27.76 Wh kg^−1^ at a maximum power density of 4000 W kg^−1^, and it still maintained a power density of 800 W kg^−1^ at a maximum energy density of 37.97 Wh kg^−1^, exhibiting the potential application of multistage core–shell composite materials in high-performance supercapacitors.

## 1. Introduction

In recent years, because of the increasingly prominent energy problem, the research on energy storage and conversion devices has attracted more and more researchers’ attention. Among all kinds of memory devices, supercapacitors are widely used for special equipment and standby energy due to their advantages of a high power density, good cycle stability, and fast charging and discharging [1].

Supercapacitors can be divided into electric double-layer capacitors (EDLCs) and pseudocapacitors (PCs). The electrodes of EDLCs are mainly made of carbon materials, which store energy mainly by the electrostatic accumulation at the interface between electrodes and electrolytes. EDLC has excellent cycle stability, but its energy density is low [2]. PC electrodes mainly consist of transition metal oxides/hydroxides/sulfides and conductive polymers. They mainly store energy through highly reversible redox reactions. Although their energy density is higher than that of EDLCs, the cyclic stability of PC electrodes is poor because of their poor structure stability during redox reactions [3]. In order to further improve the electrochemical performance of supercapacitors, asymmetric supercapacitors (ASCs) have been developed, with two electrodes using electrode materials with different energy storage mechanisms, in which one electrode used a double-layer mechanism for energy storage, and the other electrode used a reversible redox reaction for energy storage. The two different electrodes not only bring higher operating voltage to ASCs, but they also complement each other, giving ASCs excellent energy density and cycle stability while retaining a high power density [4].

Transition metal oxides/hydroxides/sulfides have received much attention for their advantages, such as high theoretical specific capacitance and the ability to perform rapid redox reactions. Compared to the corresponding transition metal oxides/hydroxides, transition metal sulfides have the advantages of relatively higher electrical conductivity, mechanical stability, thermal stability, and the ability to perform more redox reactions [5]. Moreover, transition metal sulfides, such as MnS, NiS, CoS, FeS, and CuS, have unique electrochemical properties and are widely used in the field of supercapacitors [6]. Therefore, transition metal sulfides tend to have relatively better capacitive performance. However, transition metal sulfides as supercapacitor electrode materials always face the problem of volume expansion during the charging and discharging in practical applications, which can lead to their poor cycling stability [7]. On the other hand, their poor electrical conductivity is not conducive to fast electron transport [8]. In recent years, it has been demonstrated that the combination of transition metal sulfides with carbon-based materials can effectively mitigate their structural collapse during cycling and further improve the electrochemical performance of electrode materials, as well as supercapacitors [9]. CNFs with high electrical conductivity and a large specific surface area can provide more highly conductive “landing sites” for transition metal sulfides and alleviate structural collapse during multiple charge/discharge cycles, thereby improving the electrochemical performance of the electrode materials [10,11]. Therefore, the design and fabrication of composite electrode materials are crucial for the development of high-performance supercapacitors.

**Figure 1:**
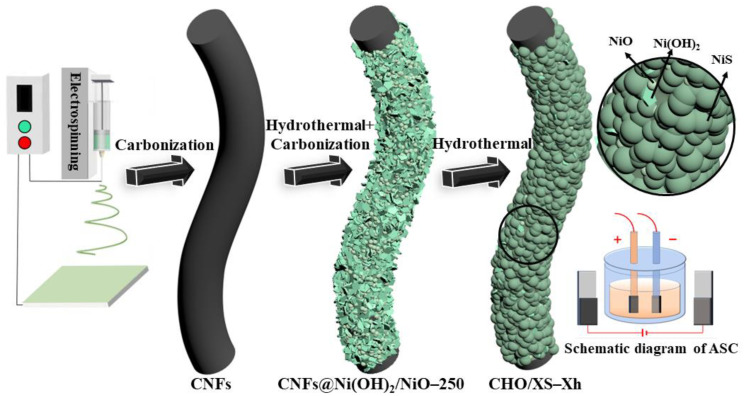
Preparation flowchart.

In this study, by combining multiple materials to compensate for each other’s shortcomings and designing their structures reasonably, a multistage core–shell composite electrode material with excellent performance for supercapacitors was fabricated. In our previous work, a composite electrode combining Ni(OH)_2_, NiO, and carbon nanofibers (CNFs) (CNFs@Ni(OH)_2_/NiO-250) was obtained which demonstrated good electrochemical properties [12]. In this work, to further enhance the electrochemical performance of CNFs@Ni(OH)_2_/NiO-250 (CHO), five transition metal sulfides (XS, X = Mn, Co, Fe, Cu, and Ni) were loaded onto its surface, using hydrothermal growth for 5 h to obtain electrode materials loaded with XS (CHO/XS-5h) and determine the optimal sample as CHO/NiS-5h. Then, the optimal hydrothermal duration was determined by investigating the effects of different hydrothermal durations (Xh, X = 1, 2, 3) on the properties of CHO/NiS-Xh. It was found that CHO/NiS-3h had the best electrochemical performance, with a specific capacitance of up to 1717 F g^−1^ (1 A g^−1^). The excellent electrochemical performance of the composite material could be attributed to its multistage core–shell structure and complementary defects of each material. The combination of multiple materials was preferred over a single material because they not only had individual advantages but also interacted to promote redox reactions. The large specific surface area of CNFs could provide carriers for the growth of Ni(OH)_2_, NiO, and NiS, and their good electrical conductivity could provide electrons for the rapid redox reactions of Ni(OH)_2_, NiO and NiS, allowing the redox reactions to proceed smoothly. Additionally, NiS acting as the outermost layer, exposed more reactive sites, thereby enhancing the overall electrochemical performance of the composite. Finally, the ASC assembled with CHO/NiS-3h as the positive electrode (CHO/NiS-3h//AC ASC) device had an energy density of 27.76 Wh kg^−1^ at a maximum power density of 4000 W kg^−1^ and maintained a power density of 800 W kg^−1^ at a maximum energy density of 37.97 Wh kg^−1^. The preparation flowchart is shown in Figure 1. 

## 2. Results and Discussion

### 2.1. Effects of Loading Different Transition Metal Sulfides on Electrode Materials

#### 2.1.1. Morphological and Structural Analysis of Electrode Materials

**Figure 2:**
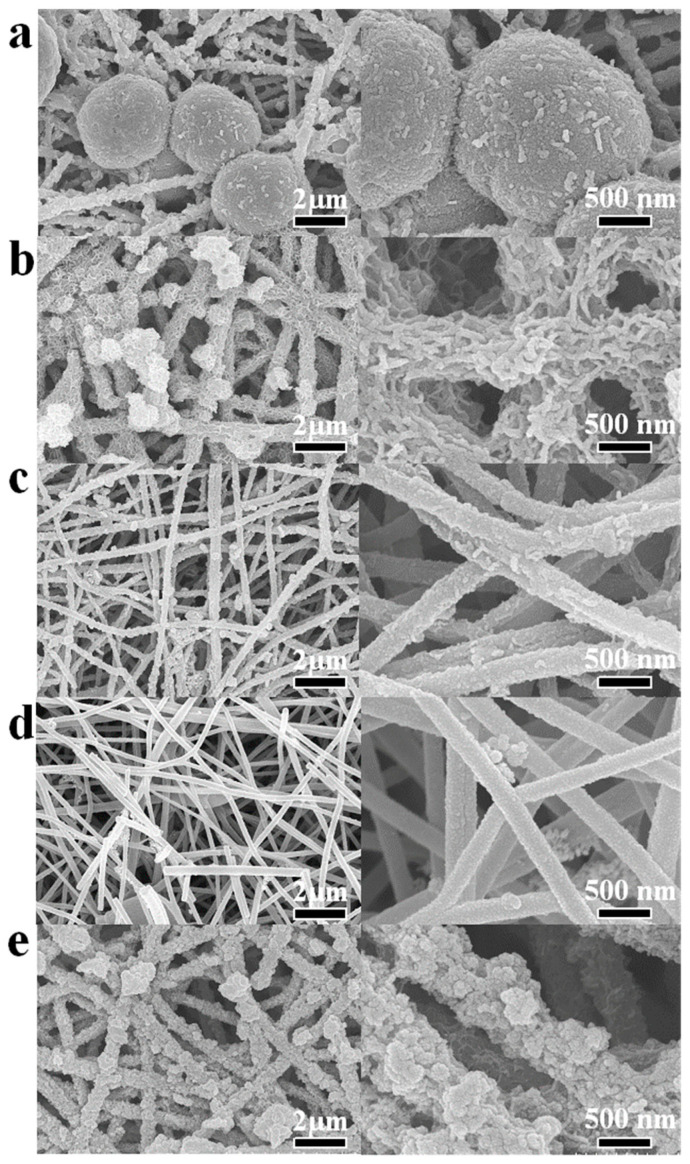
SEM images of CHO/XS-5h (the right image is a partial enlargement): (**a**) CHO/MnS-5h; (**b**) CHO/CoS-5h; (**c**) CHO/FeS-5h; (**d**) CHO/CuS-5h, and (**e**) CHO/NiS-5h.

Figure 2 shows the SEM images of CHO/XS-5h composites. It can be seen that CHO/MnS-5h (Figure 2a) had many oversized microspheres, and the diameters of the microspheres were so large that it was difficult for them to grow uniformly on the CHO surface. On the surface of CHO/CoS-5h (Figure 2b), many grooves appeared, and these might be related to the formation of CoS. The active substances loaded onto the surface of CHO/FeS-5h and CHO/CuS-5h had both fallen off (Figure 2c,d), thus indicating that FeS and CuS were not easily grown on the surface of CHO. The surface of CHO/NiS-5h (Figure 2e) was loaded with a large amount of spherical active substances of suitable size, and this might be related to the formation of NiS. Accordingly, compared to other electrode materials, CHO/NiS-5h should have relatively the most surface-active substances, making it possible to have the best electrochemical performance. 

**Figure 3:**
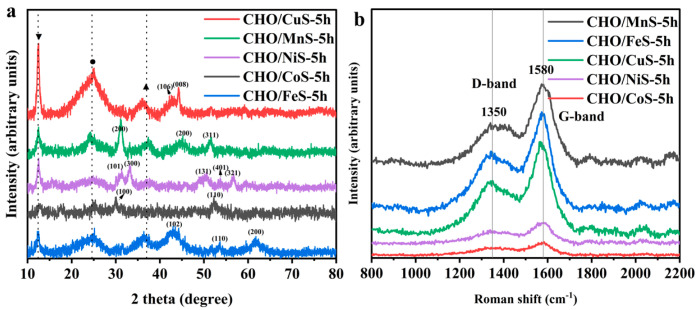
(**a**) XRD patterns and (**b**) Raman spectra of CHO/XS-5h composites.

Figure 3a shows the XRD patterns of CHO/XS-5h composites, all of which show the characteristic diffraction peaks at about 12.4°, 24.4°, and 37°, corresponding to the Ni(OH)_2_ (001) crystal plane, carbon material (002) crystal plane, and NiO (111) crystal plane, respectively. Among them, the characteristic diffraction peaks at 24.4° and 37° were less obvious because the carbon material and NiO were more covered by other materials. The characteristic diffraction peaks at about 42.9° and 44.4° in the XRD pattern of CHO/CuS-5h were associated with the (106) and (008) crystal planes of CuS (JCPDS:06-0464). The XRD pattern of CHO/MnS-5h showed the characteristic diffraction peaks at approximately 31.3°, 45.5°, and 51.2°, which were related to the (200), (220), and (311) crystal planes of MnS (JCPDS: 40-1288). The XRD pattern of CHO/NiS-5h exhibited the characteristic diffraction peaks at about 31°, 33°, 50°, 53.4°, and 56.8°, corresponding to the (101), (300), (131), (401), and (321) crystal planes of NiS, respectively (JCPDS: 65-3687). The XRD pattern of CHO/CoS-5h indicated the characteristic diffraction peaks at about 30.4° and 52.5°, which were associated with the (100) and (110) crystal planes of CoS (JCPDS: 75-0605). The XRD pattern of CHO/FeS-5h showed the characteristic diffraction peaks at approximately 43.5°, 53.6°, and 61.9°, corresponding to the (102), (110), and (200) crystal planes of FeS, respectively (JCPDS: 65-1894). Although the positions of some characteristic diffraction peaks in the XRD patterns were slightly shifted due to the excessive variety of compounds in each composite, these XRD results still proved that each transition metal sulfide was successfully grown on the surface of the corresponding composite. In addition, the characteristic diffraction peak (24.4°) corresponding to carbon materials in the XRD patterns of CHO/CuS-5h and CHO/FeS-5h were the most significant, further verifying the SEM results that the active materials (CuS and FeS) loaded onto the surface of the composites had fallen off (Figure 2c,d).

The degree of graphitization of CHO/XS-5h composites was further investigated using laser Raman testing. As shown in Figure 3, all composites had two characteristic peaks at about 1350 cm^−1^ and 1580 cm^−1^, which correspond to the D-band and G-band of the CNFs, respectively. The peak intensities of D-band and G-band of CHO/MnS-5h, CHO/CuS-5h, and CHO/FeS-5h were higher, which might be related to the less active materials on their surfaces. The peak intensity ratios (I_D_/I_G_) of CHO/MnS-5h, CHO/FeS-5h, CHO/CuS-5h, CHO/NiS-5h, and CHO/CoS-5h were calculated to be 0.48, 0.59, 0.59, 0.59, and 0.55, respectively. The I_D_/I_G_ values of CHO/FeS-5h, CHO/CuS-5h, and CHO/NiS-5h were the largest, followed by that of CHO/CoS-5h, and that of CHO/MnS-5h was the smallest. This indicated that the graphitization degree of CHO/MnS-5h was the largest, followed by that of CHO/CoS-5h, and that of CHO/FeS-5h, CHO/CuS-5h, and CHO/NiS-5h was the smallest.

Table 1 shows the atomic percentage content of each element of the CHO/XS-5h composites obtained by EDS. It could be seen that the corresponding XS was successfully grown on the surface of each composite material, among which CHO/FeS-5h and CHO/CuS-5h had less atomic content of Fe and Cu elements. This might be related to the less loading of FeS and CuS on the CHO, as this was in agreement with the results of SEM and XRD analysis.

#### 2.1.2. Electrochemical Performance of Electrode Materials

**Figure 4:**
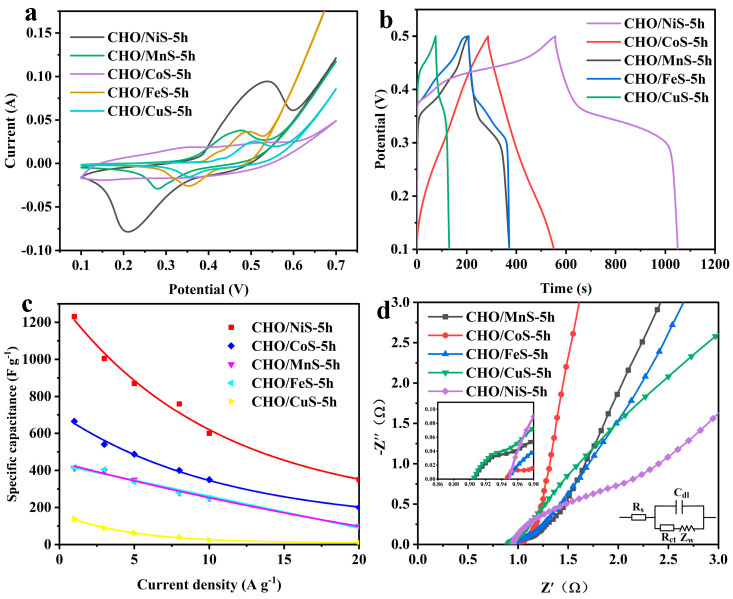
(**a**) CV curves of CHO/XS-5h composites at 5 mV s^−1^ scan rate. (**b**) GCD curves of CHO/XS-5h composites at 1 A g^−1^ current density. (**c**) Specific capacitance plots of CHO/XS-5h composites at different current densities. (**d**) Nyquist plots of CHO/XS-5h composites.

The electrochemical performances of CHO/XS-5h composites were investigated in a three-electrode system. As shown in Figure 4a, the CV curves of CHO/XS-5h at a scan rate of 5 mV s^−1^ showed that, except for CHO/CoS-5h, all other CHO/XS-5h composites had obvious redox peaks, which indicated that these composites mainly store energy through redox reactions. Among them, the CV curve of CHO/NiS-5h had the largest area, implying that CHO/NiS-5h had the highest specific capacitance. In addition, the CV curve of CHO/CoS-5h exhibited a double-layer capacitance [13]. Figure 4b shows the GCD curves of CHO/XS-5h composites at a current density of 1 A g^−1^. It can be seen that the GCD curves of CHO/NiS-5h, CHO/MnS-5h, CHO/FeS-5h, and CHO/CuS-5h all had obvious charging and discharging plateaus, and CHO/NiS-5h had the longest discharge time, indicating its optimal specific capacitance. The GCD curve of CHO/CoS-5h shows a relatively symmetrical isosceles triangle structure, which also illustrates its double-layer capacitance characteristics [13]. Figure 4c shows the specific capacitance of CHO/XS-5h composites at different current densities, and CHO/NiS-5h had the optimal specific capacitance performance, with the specific capacitance of 1231, 1005, 870, 760, 600, and 350 F g^−1^ at 1, 3, 5, 8, 10, and 20 A g^−1^, respectively.

Figure 4d exhibits the electrochemical impedance diagram of CHO/XS-5h composites, and the equivalent circuit diagram is shown in the inset. In the equivalent circuit, the intersection of the curve in the high-frequency region and the real axis represents the equivalent series resistance (Rs), the diameter of the semicircle in the high-frequency region is the charge transfer resistance (Rct), and the inclined straight line in the low-frequency region represents the ion diffusion resistance (Zw) [14,15]. From the intersection of the high-frequency region with the X-axis, it can be seen that the CHO/XS-5h composites all exhibited small Rs, and the Rs of CHO/MnS-5h and CHO/CuS-5h were smaller than those of other materials. CHO/NiS-5h had the largest Rct, followed by CHO/MnS-5h and CHO/CuS-5h, and CHO/CoS-5h and CHO/FeS-5h had the smallest Rct. In the low-frequency region, the slopes of the linear part of the curves from small to large are CHO/CoS-5h, CHO/MnS-5h, CHO/FeS-5h, CHO/CuS-5h, and CHO/NiS-5h, indicating that CHO/CoS-5h had the smallest Zw, while CHO/NiS-5h had the worst. This might be because CHO/NiS-5h was loaded with more metal sulfides with poor conductivity, resulting in its higher resistance.

In summary, CHO/NiS-5h had relatively optimal electrochemical properties, especially its extremely high specific capacitance, which was related to its good morphology, structure, and relatively abundant active substances. Therefore, CHO/NiS-5h was selected for further study.

### 2.2. Effect of Hydrothermal Reaction Duration on Electrode Materials

#### 2.2.1. Morphological and Structural Analysis of Electrode Materials

**Figure 5:**
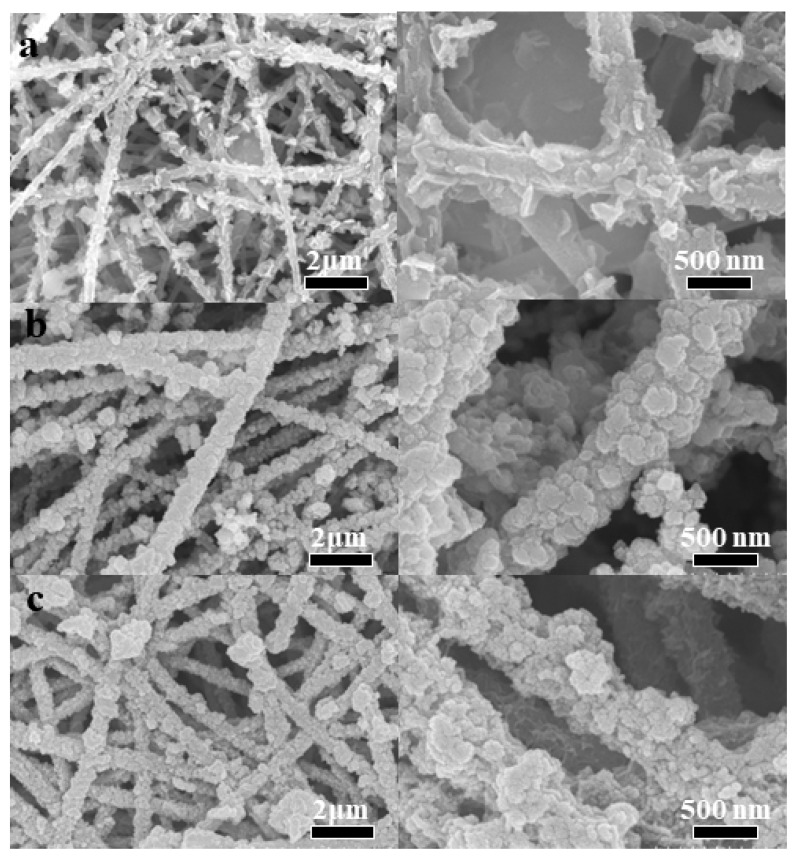
SEM images of CHO/NiS-Xh composites (the right image is a partial enlargement): (**a**) CHO/NiS-1h, (**b**) CHO/NiS-3h, and (**c**) CHO/NiS-5h.

Figure 5 shows the SEM images of CHO/NiS-Xh composites. It can be seen that NiS loaded onto the surface of CHO gradually increased with the increasing hydrothermal time. The CHO/NiS-1h surface had relatively less NiS (Figure 5a), the CHO/NiS-3h surface possessed a larger number of NiS with relatively uniform distribution (Figure 5b), and the CHO/NiS-5h surface had the largest amount of NiS but produced a buildup (Figure 5c). Table 2 demonstrats the atomic percentage content of each element of CHO/NiS-Xh obtained by EDS, which showed that the amount of S elements in CHO/NiS-Xh increased as the hydrothermal growth time continued to increase, which was caused by the continuous growth of NiS. This was in line with the findings of the SEM examination.

**Figure 6:**
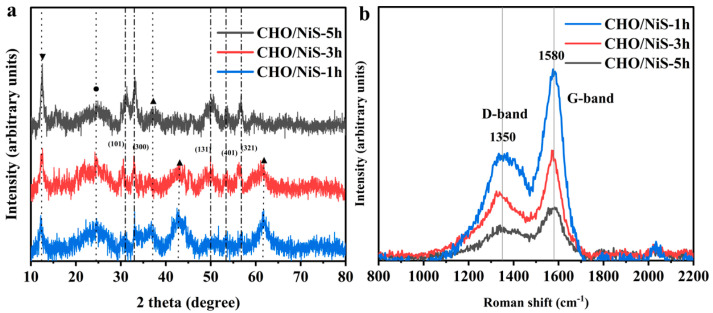
(**a**) XRD plots and (**b**) Roman plots of CHO/NiS-Xh.

Figure 6a shows the XRD patterns of CHO/NiS-Xh composites, which were similar to the XRD pattern of CHO/NiS-5h in Figure 3a. However, the characteristic diffraction peaks at about 43.1° and 62°, corresponding to the (200) and (220) crystal planes of NiO, became weaker as the hydrothermal growth time increased. In contrast, the characteristic diffraction peaks corresponding to the (101), (131), and (321) crystal planes of NiS became more and more obvious. This indicated that more and more NiS grew on the CHO surface with increasing time. This conclusion was consistent with the SEM and EDS results. The Raman spectra of the CHO/NiS-Xh composites (Figure 6b) showed that the peak intensities of the D and G bands of composites decreased with increasing hydrothermal time. This phenomenon might be brought on by an increase in NiS in the shell layer as a result of the longer hydrothermal period. The I_D_/I_G_ values of CHO/NiS-1h, CHO/NiS-3h, and CHO/NiS-5h were 0.57, 0.58, and 0.59, respectively, indicating that the graphitization degree of CHO/NiS-5h was the smallest, while that of CHO/NiS-1h was the largest.

#### 2.2.2. Electrochemical Performance of Electrode Materials

**Figure 7:**
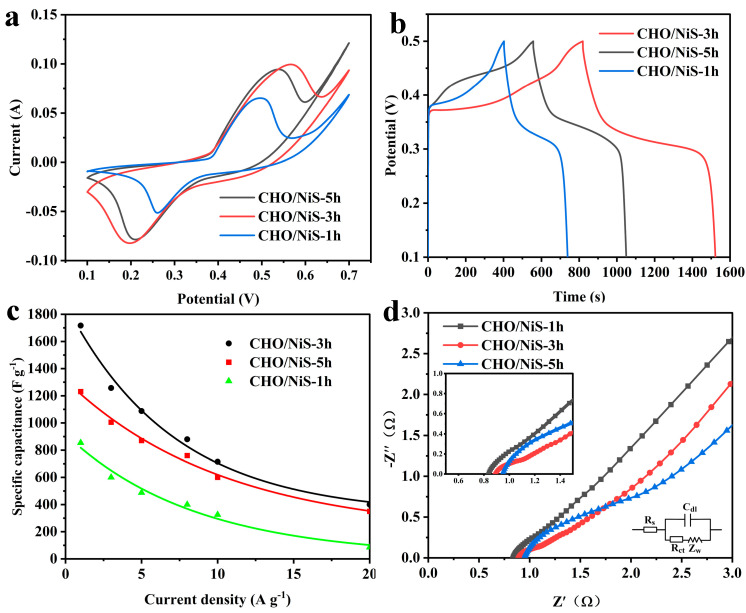
(**a**) CV curves of CHO/NiS-Xh composites at 5 mV s^−1^. (**b**) GCD curves of CHO/NiS-Xh composites at 1 A g^−1^. (**c**) Specific capacitance plots of CHO/NiS-Xh composites at different current densities. (**d**) Nyquist plots of CHO/NiS-Xh composites.

The electrochemical properties of CHO/NiS-Xh composites were tested under the three-electrode system, as shown in Figure 7. The CV curves of CHO/NiS-Xh at 5 mV s^−1^ (Figure 7a) indicated that the CV curve of CHO/NiS-3h had the largest area, illustrating that CHO/NiS-3h had the optimal specific capacitance, which might be related to its good morphology and structure. The GCD curves of CHO/NiS-Xh at 1 A g^−1^ (Figure 7b) showed that CHO/NiS-3h had the longest discharge time, indicating that CHO/NiS-3h had the optimal electrochemical properties. Figure 7c demonstrated the specific capacitance plots of CHO/NiS-Xh at different current densities, and CHO/NiS-3h had the highest specific capacitance at different current densities. Figure 7d showed that all composites had small equivalent series resistance, and the intersection point on the X-axis gradually moved to the right as the hydrothermal growth time increased, indicating that CHO/NiS-1h had the smallest Rs and CHO/NiS-5h had the largest Rs, which might be related to the increasing amount of NiS with poor conductivity. In addition, the semicircle diameter of CHO/NiS-5h was the largest and that of CHO/NiS-3h was the smallest, illustrating that CHO/NiS-3h had a superior Zw. Moreover, the slope of CHO/NiS-3h was the largest, indicating that CHO/NiS-3h had the relatively best ion diffusion mobility and fast electron transfer ability. This might be related to the moderate amount of NiS grown.

In summary, it was found that CHO/NiS-3h had the relatively optimal electrochemical performance, which was related to the size and distribution of the active substances grown on its surface being the most uniform. Therefore, it was taken as the optimal sample in the subsequent work.

### 2.3. Characterization and Application of CHO/NiS-3h 

#### 2.3.1. Morphological and Structural Analysis of CHO/NiS-3h

**Figure 8:**
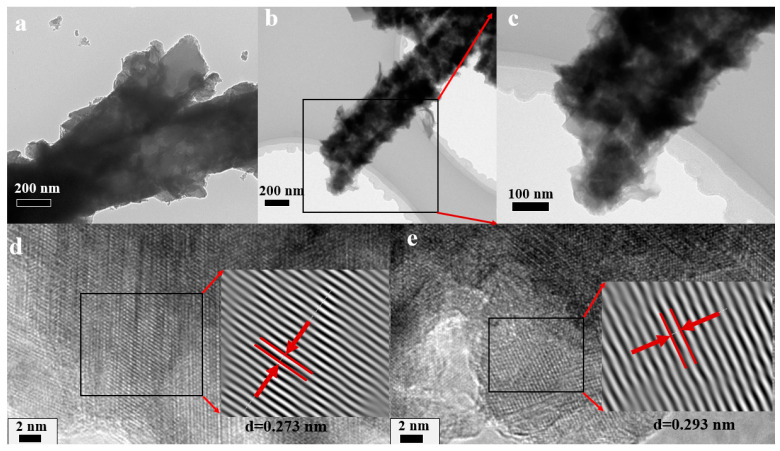
(**a**–**c**) TEM images and (**d**,**e**) lattice spacing of CHO/NiS-3h.

Figure 8 shows the TEM images of CHO/NiS-3h. From Figure 8a–c, it was clear that CHO/NiS-3h had an obvious core–shell structure, and the shell materials were uniformly distributed. On the one hand, these shell materials increased the specific surface area of the composite and the contact area between the composite and the electrolyte. On the other hand, it also brought more chemical reaction active sites, and this helped to improve the electrochemical performance of CHO/NiS-3h. Figure 8d,e illustrates the high-resolution TEM images of CHO/NiS-3h, where the lattice spacing of d = 0.273 nm and d = 0.293 nm corresponded to the (300) and (101) crystal planes of NiS, respectively, which further demonstrated the successful growth of NiS on the surface of CHO after hydrothermal reaction.

**Figure 9:**
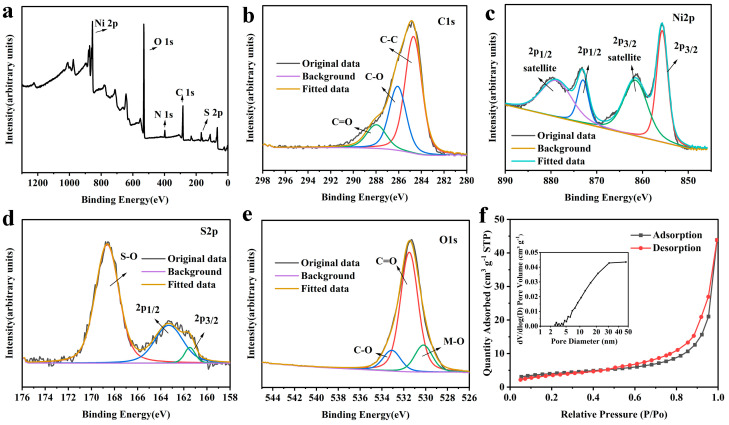
XPS patterns of CHO/NiS-3h: (**a**) full spectrum, (**b**) C1s, (**c**) Ni2p, (**d**) S2p, and (**e**) fine spectrum of O1s. (**f**) N_2_ adsorption–desorption isotherms of CHO/NiS-3h and its pore size distribution plots (inset).

The chemical composition and valence states of CHO/NiS-3h were investigated by XPS, as shown in Figure 9. The full XPS spectrum of CHO/NiS-3h (Figure 9a) showed that it contained five elements, C, N, O, Ni, and S, and this was consistent with the EDS results. The XPS spectrum of C1s (Figure 9b) could be divided into three characteristic peaks, namely C–C (284.7 eV), C–O (286.1 eV), and C=O (287.9 eV). In the XPS pattern of Ni2p (Figure 9c), the characteristic peaks at 855.7 eV and 872.9 eV binding energies were associated with the spin–orbit peaks of Ni2p 3/2 and Ni2p 1/2, respectively, and the characteristic peaks at 861.6 eV and 879.0 eV binding energies were related to the satellite peaks of the Ni2p 3/2 and Ni2p 1/2 spin–orbit peaks, respectively. In the XPS patterns of S2p (Figure 9d), the characteristic peaks at binding energies of 163.3 eV and 161.5 eV could be attributed to the S2p 3/2 and S2p 1/2 spin–orbit peaks, while the characteristic peak at binding energy of 168.6 eV could be attributed to the S–O bond. The O1s XPS pattern (Figure 9e) could also be divided into three characteristic peaks, namely C–O (533.0 eV), C=O (531.5 eV), and M–O (529.9 eV).

Figure 9f showed the N_2_ adsorption–desorption isotherms of CHO/NiS-3h and its pore size distribution, which revealed that its pore structure was primarily composed of mesopores and macropores. The mesopores and macropores were beneficial for composites to fully contact with the electrolyte, promoting the rapid ion transport and improving the electrochemical properties of composites. Table 3 showed the specific surface area and pore size distribution of CHO/NiS-3h, indicating a BET specific surface area of 12.3815 m^2^ g^−1^ and a total pore volume of 0.063 cm^3^ g^−1^.

#### 2.3.2. Electrochemical Performance of CHO/NiS-3h 

**Figure 10:**
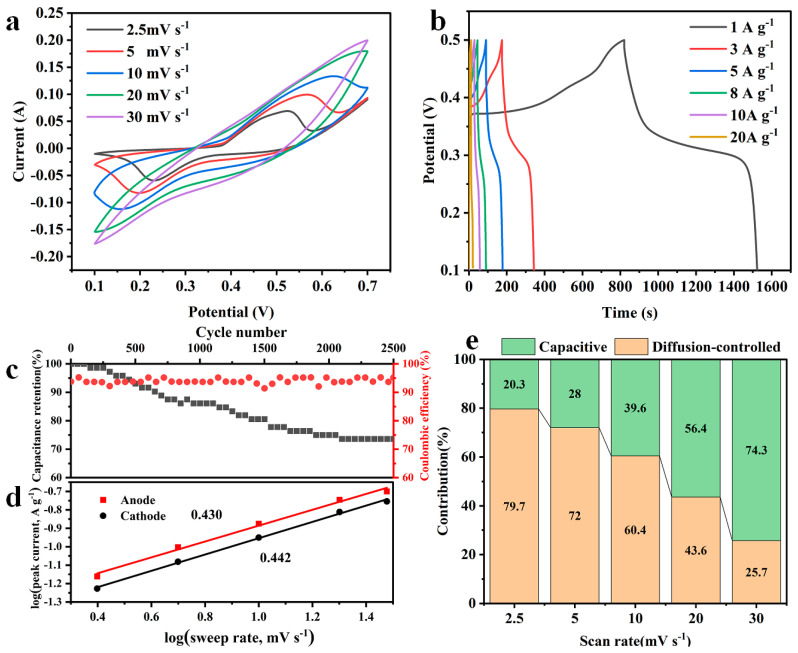
(**a**) CV curves of CHO/NiS-3h at different sweep rates. (**b**) GCD curves of CHO/NiS-3h at different current densities. (**c**) Cycling stability and coulomb efficiency of CHO/NiS-3h at 3 A g^−1^. (**d**) Relationship between log(v) and log(ip). (**e**) Histogram of capacitance contribution at different sweep rates.

The CV curves of CHO/NiS-3h at different sweep rates (Figure 10a) showed that there were obvious redox peaks in the CV curves at lower scanning rates, and with the increase of the sweep rate, the redox peaks shifted regularly and kept moving toward the high potential, resulting in the weakening of redox peaks. This might be related to the high sweep rate, which prevented the active material from reacting in time. The GCD curves of CHO/NiS-3h at different current densities (Figure 10b) all exhibited good symmetry, indicating that CHO/NiS-3h could undergo a relatively highly reversible redox reaction during the charge and discharge process. The Faraday redox reaction of NiS is shown in Equation (1):

(1) $$\mathrm{NiS}+{\mathrm{OH}}^{-}↔\mathrm{NiOH}+e^{-}$$

The specific capacitances of CHO/NiS-3h at 1, 3, 5, 8, 10, and 20 A g^−1^ were 1717, 1257.5, 1087.5, 880, 715, and 400 F g^−1^, respectively. Compared with other reported NiS-based electrode materials, CHO/NiS-3h still had good electrochemical performance (Table 4). Figure 10c indicated that CHO/NiS-3h had 73.6 ± 2% capacitance retention and 94.5 ± 2% coulomb efficiency after 2500 cycles at 3 A g^−1^, illustrating that CHO/NiS-3h had better cycling stability and coulomb efficiency at 3 A g^−1^.

Furthermore, based on its CV curves at different scan rates, the charge storage mechanism of CHO/NiS-3h was further investigated to determine whether it is dominated by the contribution of the diffusion control process or the contribution of the capacitance. Equations (2) and (3) were used to find the relationship between scan rate (v) and peak current (i_p_) in the CV curves [23,24].

(2) $$i_{p}={\mathrm{av}}^{b}$$

(3) $$\log\left(i_{p}\right)=\mathrm{blog}\left(v\right)+\log\left(a\right)$$

where a and b are adjustable parameters. 

The charge storage mechanism of electrodes is represented by the b-value, which is typical for determining the contribution of the diffusion control process (b = 0.5) and the contribution of the capacitance (b = 1). According to Equations (2) and (3), the slopes of the linear plots composed of log(v) and log(i_p_) could be obtained for the oxidation and reduction peaks with b-values of 0.430 and 0.442, respectively (Figure 10d). It could be inferred that the diffusion-controlled process of the CHO/NiS-3h composite dominated the charge energy storage mechanism. The percentages of diffusion control process contribution and capacitance contribution could be obtained according to Equation (4) [25,26]:

(4) $$i=k_{1}v+k_{2}v^{0.5}$$

where i denotes the current; and k_1_v and k_2_v^0.5^ denote the capacitance and diffusion-controlled processes, respectively.

Figure 10e exhibited the contribution percentage plots of capacitance and diffusion control processes at different scan rates. It could be concluded that the contribution of the diffusion control process dominates at the sweep rate of 2.5 mV s^−1^, and the capacitance contribution gradually increases and dominates as the sweep rate increases to 30 mV s^−1^. It could be related to the instability of the NiS structure at high scan rates and the untimely occurrence of its redox reaction.

#### 2.3.3. Electrochemical Performance of Asymmetric Supercapacitors

**Figure 11:**
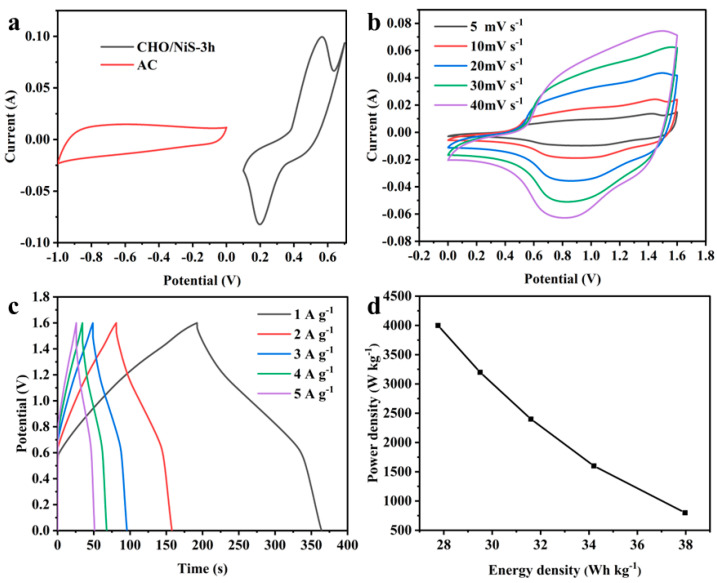
(**a**) CV curves of CHO/NiS-3h and AC in the three-electrode system at 5 mV s^−1^. (**b**) CV curves of ASC at different scan rates. (**c**) GCD curves of ASC at different current densities. (**d**) Energy density and power density curves.

CHO/NiS-3h//AC ASC was assembled with CHO/NiS-3h as the positive electrode and AC as the negative electrode, and its performance was investigated, as illustrated in Figure 11. Figure 11a showed the CV curves of two electrodes in ASC at a 5 mV s^−1^ scan rate in the three-electrode system, and these were used to determine the operating voltage window of CHO/NiS-3h//AC ASC. As expected, the stable electrochemical window of ASC could be extended to 1.6 V. Meanwhile, referring to the operating voltage windows of other nickel-based supercapacitors, 1.6 V was finally chosen as the voltage window of the ASC device to maximize the electrochemical performance of ASC [27,28]. The CV curve shape of ASC at different scan rates did not change much with the increase of the scan rate (Figure 11b), and the GCD curve shape of ASC at different current densities was also almost constant as the current density increased (Figure 11c), meaning that the ASC device had good electrochemical reversibility. The calculated specific capacitances of CHO/NiS-3h//AC ASC at 1, 2, 3, 4, and 5 A g^−1^ were 106.8, 96.3, 89, 83, and 78.1 F g^−1^, respectively. Figure 11d shows the energy density and power density relationship curves of ASC, from which it could be concluded that ASC had an energy density of 27.76 Wh kg^−1^ at a maximum power density of 4000 W kg^−1^, and it still had a power density of 800 W kg^−1^ at a maximum energy density of 37.97 Wh kg^−1^, which still had a good performance compared with the reported ASCs assembled with NiS-based composites (Table 5).

## 3. Materials and Methods

### 3.1. Materials

Polyacrylonitrile (PAN, Mw = 150,000) was purchased from Hefei Sipin Technology Co., Ltd. (Hefei, China). Urea was supplied by Beijing Inoke Technology Co., Ltd. (Beijing, China). Ni(CH_3_COO)_2_·4H_2_O was provided by Utop Technology Suzhou Co., Ltd. (Suzhou, China). Mn(CH_3_COO)_2_·4H_2_O, Ni(NO_3_)_2_·6H_2_O and Cu(CH_3_COO)_2_·H_2_O were supplied by Shanghai Aladdin Company (Shanghai, China). Co(CH_3_COO)_2_·4H_2_O, Fe(CH_3_COO)_3_ and CH_4_N_2_S were purchased by Jiangsu Argon Krypton Xenon Company (Shanghai, China). Activated carbon (AC) was provided by Youtepu technology Suzhou Co., Ltd. (Suzhou, China). Polytetrafluoroethylene emulsion (PTFE, 60 wt%) was supplied from Shanghai Rin Technology Development Co., Ltd. (Shanghai, China). (CH_2_OH)_2_, N,N-Dimethylformamide (DMF), and ethanol were obtained from Jiangsu Qiangsheng Functional Chemical Co., Ltd. (Suzhou, China). All reagents are of analytical grade, and the water used was deionized water.

### 3.2. Characterization

The morphology of the samples was observed using an ultra-high resolution field emission scanning electron microscope (HR-FESEM and S-4700, Hitachi, Ltd., Tokyo, Japan) and a field emission transmission electron microscope (FETEM, TecnaiG2F20, FEI Company, Portland, OR, USA). Elemental contents and energy spectra were measured using TM 3030 and Regulus 8100 (Hitachi, Ltd., Tokyo, Japan), while the crystal structures of samples were analyzed through X-ray diffraction (XRD, D8 Advance, Karlsruhe, Germany, CuKα, scanning speed 2° min^−1^, and 2Theta = 5–80°) and Raman spectroscopy (Raman, Jobin Yvon, Paris, France, 532 nm laser). The specific surface area and pore size distribution of the samples were characterized with a four-station automatic physical adsorption instrument (ASAP 2460, Micrometrics, Norcross, GA, USA). The chemical compositions of the samples were measured using X-ray photoelectron spectroscopy (XPS, Thermo Scientific K-Alpha, Manchester, UK; voltage, 12 kV; current, 6 mA).

### 3.3. Preparation of CHO

First, 4 g of PAN was weighed and dissolved in 38 g of DMF solution. Then, 0.72 g (4 mmol) of Ni(CH_3_COO)_2_ was weighed and dissolved in the above solution to increase the electrical conductivity of the composite [29]. The spinning precursor solution was obtained when the solute was completely dissolved. The composite nanofiber membranes (CNFMs) were prepared using the electrospinning method, with temperature control at about 25 °C, humidity control at about 50%, a flow rate of 1 mL h^−1^, a receiving distance of 18 cm, and a spinning voltage of 16 kV.

Then, the CNFMs were pre-oxidized in a muffle furnace at 250 °C for 2 h in an air atmosphere with a heating rate of 2 °C min^−1^. Afterwards, the pre-oxidized samples were carbonized in a tube furnace at 800 °C for 2 h in a N_2_ atmosphere, with a heating rate of 5 °C min^−1^, followed by hydrothermal growth of Ni(OH)_2_. The growth solution consisted of 80 mL of deionized water, 1.16 g of Ni(NO_3_)_2_·6H_2_O, and 2.4 g of urea. The hydrothermal reaction temperature was 120 °C, and the reaction time was 6 h. Finally, the samples fabricated were subjected to low-temperature carbonization in a N_2_ atmosphere at 250 °C for 1 h, with a heating rate of 5 °C min^−1^, thus obtaining the CHO composite. The preparation method was also described in our previously reported paper [12,30].

### 3.4. Growth of Transition Metal Sulfide 

Different transition metal sulfides were grown on CHO, using a hydrothermal process. In total, 2.5 mmol of thiourea and 3.5 mmol of acetate (M(CH_3_COO)_X_, (M = Mn, Ni, Co, Fe, Cu. X = 2, 3) were weighed and dissolved in 50 mL ethylene glycol and then stirred at room temperature for 6 h to dissolve completely. Afterwards, the solution was transferred to a 100 mL hydrothermal reactor, and a certain amount of CHO was added and left for 12 h. Then, the hydrothermal reactor was placed in a blast drying oven at 180 °C for 5 h. Finally, the composites after hydrothermal growth were removed, washed repeatedly with ethanol and deionized water, and dried at 60 °C for 6 h. The final obtained materials were noted as CHO/XS-5h (X = Mn, Ni, Co, Fe, and Cu). When NiS-loaded composites were prepared, the hydrothermal reaction durations were changed to 3 h and 1 h, respectively, and the obtained materials were recorded as CHO/NiS-Xh (X = 1, 3, 5).

### 3.5. Preparation of Working Electrodes

Firstly, the nickel foam was sheared into 1 × 2 cm rectangular strips, followed by sonication with 1 M hydrochloric acid solution for 0.5 h, and then it was sonicated using anhydrous ethanol for 0.5 h. Finally, it was dried and set aside. The prepared composites were dried in an oven and then ground into fine powder, followed by weighing the obtained powder (active substance), carbon black (conductive agent), and PTFE dispersion (binder) in the ratio of 8:1:1, and they were mixed into a uniform slurry by adding a small amount of ethanol dropwise. Afterwards, the slurry was applied to the area of 1 × 1 cm of nickel foam with a scraper, and the nickel foam was dried under an atmosphere of 60 °C for 12 h. After drying, the nickel foam was pressed under the pressure of 10 MPa for about 10 s. The working electrode preparation was completed, and the final mass of the coated substances was 5 mg. 

### 3.6. Assembly of Asymmetric Supercapacitors

CHO/NiS-3h was used as the positive electrode, AC as the negative electrode, and 3 M KOH solution as the electrolyte; the two electrodes were assembled in an electrolytic cell to form a CHO/NiS-3h//AC ASC. The positive and negative electrodes of the ASC should follow the principle of q+ = q−, and their masses should be calculated by Equation (5):

(5) $$\frac{m_{+}}{m_{-}}=\frac{C_{-}\times {\Delta V}_{-}}{C_{+}\times {\Delta V}_{+}}$$

where + and − represent the positive and negative electrodes, respectively; m represents the mass of the active substance (mg); C represents the specific capacitance (F g^−1^); and ΔV represents the range of the voltage window (V).

### 3.7. Electrochemical Property Characterization

The electrochemical performance of a single electrode was tested in a three-electrode system, using a CHI660E electrochemical workstation, in which a platinum sheet electrode served as the auxiliary electrode, an HgO/Hg electrode served as the reference electrode, a clamped nickel foam electrode served as the working electrode, and a 3 M KOH solution served as the electrolyte. The formula for calculating the specific capacitance of the tested composite is shown in Equation (6):

(6) $$C=\frac{I\times \Delta t}{m\times \Delta V}$$

where I is the current (A), and Δt is the discharge time (s).

The electrochemical properties of ASC were tested in a two-electrode system, and its specific capacitance, energy density, and power density were calculated according to the following (Equations (7)–(9)):

(7) $$C=\frac{I\times \Delta t}{M\times \Delta V}$$

(8) $$P=3600\times \frac{E}{\Delta t}$$

(9) $$E=\frac{1}{2}\times \frac{1}{3.6}\times C\times {\Delta V}^{2}$$

where M is the mass sum of positive and negative active substances (mg), V is the operating voltage window of supercapacitors (V), t is the discharge time (s), E is the energy density (Wh kg^−1^), and P is the power density in (W kg^−1^).

## 4. Conclusions

In this study, the composite electrode materials with multiple core–shell structures were obtained by further hydrothermal growth of different transition metal sulfides (MnS, NiS, CoS, FeS, and CuS) on CHO. The effects of these five loaded transition metal sulfides on the electrochemical properties of electrodes were investigated, and the electrode material with relatively optimal performance was determined to be the NiS-loaded electrode material. On this basis, the optimal duration for hydrothermal growth of NiS on CHO was confirmed to be 3 h by discussing the electrochemical properties of electrode materials loaded with NiS obtained at different hydrothermal durations. It was found that the optimal sample obtained (CHO/NiS-3h) had the highest specific capacitance, with a specific capacitance of up to 1717 F g^−1^ at 1 A g^−1^. Finally, the ASC device assembled with CHO/NiS-3h (CHO/NiS-3h//AC ASC) showed excellent electrochemical performances, with an energy density of 27.76 Wh kg^−1^ at a maximum power density of 4000 W kg^−1^, and still 800 W kg^−1^ at a maximum energy density of 37.97 Wh kg^−1^.

## Figures and Tables

**Table 1:** Table of elemental contents of CHO/XS-5h.

Samples	Elemental Content of Each Sample (Atomic %)								
	C	N	O	Ni	Mn	Co	Fe	Cu	S
CHO/MnS-5h	53.49	6.30	20.37	7.34	9.61	\	\	\	2.89
CHO/CoS-5h	67.78	7.39	13.63	3.70	\	3.09	\	\	4.41
CHO/FeS-5h	79.15	7.50	6.66	2.72	\	\	0.71	\	3.26
CHO/CuS-5h	86.51	4.42	4.33	3.03	\	\	\	0.22	1.49
CHO/NiS-5h	59.87	7.15	15.07	14.96	\	\	\	\	2.95

**Table 2:** Elemental contents of CHO/NiS-Xh.

Samples	Elemental Content of Each Sample (Atomic %)				
	C	N	O	Ni	S
CHO/NiS-5h	59.87	7.15	15.07	14.96	2.95
CHO/NiS-3h	47.96	12.72	27.07	10.38	1.86
CHO/NiS-1h	58.87	11.75	24.05	8.44	1.09

**Table 3:** Information on specific surface area and pore size distribution of CHO/NiS-3h.

S_A_ (m^2^ g^−1^)	V_T_ (cm^3^ g^−1^)	V_S_ (cm^3^ g^−1^)	V_L_ (cm^3^ g^−1^)	W_avg_ (nm)
12.3815	0.068	0.003	0.065	12.845

S_A_, specific surface area; V_T_, total pore volume; V_L_, volume of pores with a pore diameter of 2.5–300 nm; V_S_, volume of pores with pore size less than 2.5 nm, calculated as V_T_ − V_L_; W_avg_, average pore width at desorption (nm).

**Table 4:** Comparison of CHO/NiS-3h with other nickel-based materials.

Electrode Material	Preparation Method	Morphology	Specific Capacitance (F g^−1^)	Electrolyte	Ref.
CHO/NiS-3h	Electrospinning, carbonization, hydrothermal growth	Core–shell fibers	1717 (1 A g^−1^)	3M KOH	This work
NiS	Hydrothermal growth	Multistage NiS micro flower	1122.7 (1 A g^−1^)	3 M KOH	[6]
NiS/Cu_7_S_4_-DT	Hydrothermal growth	Nanoparticles	1674 (1 A g^−1^)	6 M KOH	[4]
Co-Ni-S@CoNi-LDH	Hydrothermal growth	Core–shell nanosheet array	2414 (1 A g^−1^)	3 M KOH	[16]
α-NiS@MWCNT	Hydrothermal growth, muffle furnace heating	Microsphere	2057 (1 A g^−1^)	2 M KOH	[17]
MoS_2_/NiS	Hydrothermal growth	Egg yolk shell microsphere	1194 (1 A g^−1^)	6 M KOH	[18]
NiS-3D-Nf	Hydrothermal growth	Granular NiS	770 (1 A g^−1^)	3 M KOH	[19]
NiS/rGO	Microwave-hydrothermal method	Granular hybrid	1745.67 (1 A g^−1^)	2 M KOH	[20]
NiS@CoS	Hydrothermal growth, electrodeposition	Core–shell structure	1210 (1 A g^−1^)	2 M KOH	[21]
Ni*_x_*S*_y_*–TRGO	Hydrothermal growth	Microflorate	1602.2 (1 A g^−1^)	2 M KOH	[22]

**Table 5:** Comparison of CHO/NiS-3h//AC ASC with the reported ASCs assembled with NiS-based composites.

Assembly Materials	Specific Capacitance (F g^−1^)	Maximum Power Density (W kg^−1^)	Energy Density at Maximum Power Density (Wh kg^−1^)	Voltage Window (V)	Ref.
CHO/NiS-3h//AC	106.8 (1 A g^−1^)	4000	27.76	1.6	This work
NiS//AC	69.1 (1 A g^−1^)	8800	12.9	1.8	[6]
NiS/Cu_7_S_4_-DT//AC	157 (0.5 A g^−1^)	7492.5	20.8	1.5	[4]
Co-Ni-S@CoNi-LDH//AC	147.27 (1 A g^−1^)	8500.93	39.91	1.7	[16]
α-NiS@MWCNT//AC	80 (1 A g^−1^)	2175	12	1.5	[17]
N-NiS//PCN	109 (1 A g^−1^)	10,900	19.99	1.6	[27]
Ni*_x_*S*_y_*–TRGO//TRGO	123.7 (1 A g^−1^)	7500	23.75	1.5	[22]
NiS/CNFs-2//AC	29.8 mAh g^−1^ (1 A g^−1^)	7500	12.69	1.6	[28]
NiS@CoS//AC	75.9 (1 A g^−1^)	3849	6.875	1.5	[21]

## Data Availability

Data is contained within the article.
